# Perceptions in 3.6 Million Web-Based Posts of Online Communities on the Use of Cancer Immunotherapy: Data Mining Using BERTopic

**DOI:** 10.2196/60948

**Published:** 2025-02-10

**Authors:** Xingyue Wu, Chun Sing Lam, Ka Ho Hui, Herbert Ho-fung Loong, Keary Rui Zhou, Chun-Kit Ngan, Yin Ting Cheung

**Affiliations:** 1 School of Pharmacy, Faculty of Medicine The Chinese University of Hong Kong Hong Kong SAR China; 2 Department of Clinical Oncology Faculty of Medicine The Chinese University of Hong Kong Hong Kong SAR China; 3 Data Science Program Worcester Polytechnic Institute Worcester, MA United States

**Keywords:** social media, cancer, immunotherapy, perceptions, data mining, oncology, web-based, lifestyle, therapeutic intervention, leukemia, lymphoma, survival, treatment, health information, decision-making, online community, machine learning

## Abstract

**Background:**

Immunotherapy has become a game changer in cancer treatment. The internet has been used by patients as a platform to share personal experiences and seek medical guidance. Despite the increased utilization of immunotherapy in clinical practice, few studies have investigated the perceptions about its use by analyzing social media data.

**Objective:**

This study aims to use BERTopic (a topic modeling technique that is an extension of the Bidirectional Encoder Representation from Transformers machine learning model) to explore the perceptions of online cancer communities regarding immunotherapy.

**Methods:**

A total of 4.9 million posts were extracted from Facebook, Twitter, Reddit, and 16 online cancer-related forums. The textual data were preprocessed by natural language processing. BERTopic modeling was performed to identify topics from the posts. The effectiveness of isolating topics from the posts was evaluated using 3 metrics: topic diversity, coherence, and quality. Sentiment analysis was performed to determine the polarity of each topic and categorize them as positive or negative. Based on the topics generated through topic modeling, thematic analysis was conducted to identify themes associated with immunotherapy.

**Results:**

After data cleaning, 3.6 million posts remained for modeling. The highest overall topic quality achieved by BERTopic was 70.47% (topic diversity: 87.86%; topic coherence: 80.21%). BERTopic generated 14 topics related to the perceptions of immunotherapy. The sentiment score of around 0.3 across the 14 topics suggested generally positive sentiments toward immunotherapy within the online communities. Six themes were identified, primarily covering (1) hopeful prospects offered by immunotherapy, (2) perceived effectiveness of immunotherapy, (3) complementary therapies or self-treatments, (4) financial and mental impact of undergoing immunotherapy, (5) impact on lifestyle and time schedules, and (6) side effects due to treatment.

**Conclusions:**

This study provides an overview of the multifaceted considerations essential for the application of immunotherapy as a therapeutic intervention. The topics and themes identified can serve as supporting information to facilitate physician-patient communication and the decision-making process. Furthermore, this study also demonstrates the effectiveness of BERTopic in analyzing large amounts of data to identify perceptions underlying social media and online communities.

## Introduction

In recent years, immunotherapy has emerged as one of the most promising therapeutic approaches for treating cancer. It works by activating the innate immune system to identify and attack cancer cells. Immunotherapy encompasses various strategies, including immune checkpoint inhibitors, T-cell transfer therapy, monoclonal antibodies, treatment vaccines, and immune system modulators [1]. These immunotherapeutic strategies have received approval for the treatment of several cancer types such as lung cancer, prostate cancer, chronic lymphocytic leukemia, and non-Hodgkin lymphoma [2]. Immunotherapy offers substantial benefits in terms of precision, specificity, and long-term survival improvements, representing a significant breakthrough in cancer treatment [3]. 

Immunotherapy has made remarkable progress and demonstrated clinical value. However, there are drawbacks that may hinder its clinical use and acceptance by patients with cancer. One notable limitation is the variability in individuals’ responses to immunotherapy. Although the treatment may be effective in some patients with specific types of cancers, it may not be as effective for others with the same cancer types [4]. Besides, immunotherapy-related adverse events (irAEs) have been observed with the increasing frequency and duration of immunotherapy usage. Patients’ decisions to undergo immunotherapy are influenced by a range of factors, including their perceptions of its efficacy, side effects, procedural aspects, costs, their levels of knowledge about the treatment, and the comprehensiveness of advice provided by health care providers [5,6]. These findings highlight the importance of understanding patients’ perspectives regarding immunotherapy to manage the uncertainties faced by patients and to make informed decisions on whether to proceed with immunotherapy.

The internet has become an indispensable source of health information for patients [7]. Many patients scour the internet for medical guidance, share their personal experiences, and interact on social media platforms [8]. The internet, therefore, provides a valuable platform for capturing diverse perspectives. Several studies have explored these perspectives by collecting data from social media platforms and forums [9-13]. For example, through analyzing posts from social media, a study revealed that pain and fatigue were the most commonly discussed symptoms among patients with non–small-cell lung cancer regarding the use of immunotherapy [10]. Another study found that 55% of patients’ posts and 37% of caregivers’ posts expressed positive perceptions of immunotherapy for treating advanced bladder cancer [11]. Notably, these previous studies usually extracted posts from a single site and analyzed only a small portion of the available posts. Moreover, these studies primarily focused on patients with specific cancer types, and thus, the findings may not be generalizable to patients with other cancer types for which immunotherapy has been approved [12]. 

One challenge in analyzing data from the internet and social media posts is dealing with vast amounts of unstructured text, which necessitates extensive preprocessing measures prior to analysis. Different machine learning techniques have been explored to increase the efficiency of online text analysis. BERTopic, a topic modeling technique that is an extension of the Bidirectional Encoder Representation from Transformers (BERT) machine learning model, has been used to identify the underlying public perceptions within social media posts. It has demonstrated the capability to unveil latent patterns and extract topics from large datasets [14-16]. Compared to other models, BERT models provide a stronger understanding of the contextual meaning of each word in document representations, which is attributable to their bidirectional training of transformers [17]. This enables the generation of more accurate topics than those generated using traditional statistical models. Moreover, the BERT model is continuously trained and updated by researchers, ensuring ongoing improvements [18]. However, no study has employed this technique to explore the viewpoints of the online cancer community on immunotherapy.

With the objective of understanding the perspectives of online cancer communities regarding immunotherapy, this study used BERTopic to analyze a large number of posts extracted from multiple social media and online forums. The findings of this study may offer valuable insights for clinicians, patients, and researchers to enhance decision-making processes when considering immunotherapy as a treatment option.

## Methods

### Overview

**Figure 1:**
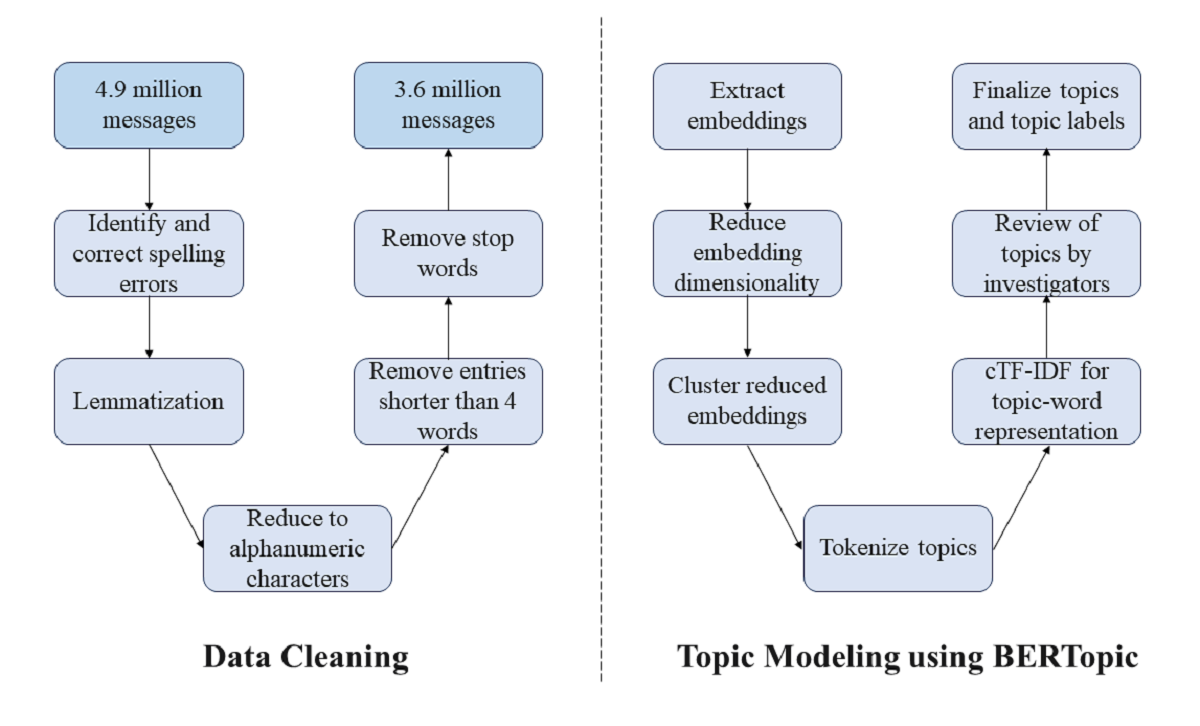
Flowchart depicting the data mining process. BERTopic: a topic modeling technique that is an extension of the Bidirectional Encoder Representation from Transformers; cTF-IDF: class-based term frequency-inverse document frequency.

This was a retrospective study that involved analyzing texts collected from social media platforms and online forums. The study proceeded through 4 primary methodological phases: (1) collecting textual data, (2) cleaning and processing the extracted texts, (3) performing and optimizing topic modeling, and (4) conducting thematic analysis. The study process workflow is illustrated in Figure 1.

### Ethics Approval

This study exclusively utilizes anonymized data from publicly available sources. The quotes presented in this report are modified or paraphrased to prevent any potential identification and direct linkage to the original posts/quotes. All identifiers or pseudo identifiers have been removed from the report. This study was approved by the Survey and Behavioral Research Ethics Committee of the Chinese University of Hong Kong (reference SBRE-20-864).

### Data Sources and Collection

For this study, textual data were collected from online forums and social media platforms. For consistency and minimal translation concerns, only English-language texts were included. The posts from these platforms dated before November 15, 2022, were included, with varying start dates. For social media platforms, posts were extracted from Facebook, Twitter, and Reddit. These platforms were chosen for their popularity and the substantial textual content they contain [19]. For cancer-related online forums, the selection criteria have been detailed in a previous study [20]. To describe briefly, we first used the top search engines (Google, Microsoft Bing, Yahoo, and Yandex) to search for health forums by using different combinations of search terms (cancer, tumor/tumour, patient, and forum) [21]. The search results were extracted and examined by the investigators. Web forums were included if they (1) appeared in more than one search result, (2) were open access (ie, no membership or passwords were needed to access the messages), (3) were active for at least the past 5 years, (4) had at least 10 messages posted to the group within the past 30 days from the date of the search, and (5) enabled web scraping of posts or feeds using Python (Python Software Foundation) or R (R Foundation for Statistical Computing). Finally, 16 different online cancer-related forums, including Cancer Chat, Cancer Survivors Network, Cancer Council Online Community, and other forums, were included for the analysis (Table 1). Search terms related to immunotherapy were identified from multiple authoritative websites, including the American Cancer Society, Cancer Research UK, and the National Cancer Institute (Table S1 in Multimedia Appendix 1). Site-provided search engines were utilized to identify related posts.

**Table 1:** List of the online health forums selected for data extraction [22-37].

Name of web forum	Forum start date^a^
Cancer Chat [22]	2008
Jo's Cervical Cancer Trust Forum [23]	2004
Prostate Cancer UK [24]	2014
Bowel Cancer UK Community [25]	2015
Cancer Survivors Network [26]	2000
Breastcancer.org community [27]	2004
Pancreatic Cancer UK Forum [28]	2007
Breast Cancer Now Forum [29]	2005
Cancer Council Online Community [30]	2009
Irish Cancer Society Community [31]	2008
Navigating Care [32]	2009
TC Cancer.com [33]	2004
HealthBoards (cancer) [34]	2000
Melanoma Patient Forum [35]	2010
Macmillan Cancer Support [36]	2008
HealthUnlocked [37]	2013

^a^In cases where the start dates of certain online health forums were not explicitly provided, an estimation was made based on the published date of the first post available.

### Data Preprocessing

Spark NLP, a natural language processing library for Python built on top of Apache Spark, was utilized to preprocess the textual data [38]. The texts extracted from the forums and social media sites were subjected to a thorough cleaning process to enhance their suitability for further analysis. The cleaning process involved multiple steps, including checking and correcting spelling errors, lemmatization, removal of stop words, and elimination of short entries (Figure 1). Each text message was first spell-checked, and the vocabulary was reduced to its base root form by using Spark NLP. After that, each word vocabulary was reduced to alphanumeric characters that contained only letters from “a to z,” “A to Z,” and “0 to 9.” For a precise and semantically meaningful text corpus, any message composed of fewer than 4 words as well as commonly occurring words (eg, the, a, and, in) that carried little or no meaning in the message were removed. Finally, the cleaned text messages were processed using the medical language models from Healthcare Spark NLP, a pretrained pipeline to recognize medical terminologies within the messages [39].

### Topic Modeling

The next step involved the extraction of topics from the data. Topic modeling, an unsupervised machine-learning approach, was employed to unveil hidden semantic structures and extract distinct topics from the extracted textual data [40]. In this study, we first evaluated 10 common topic modeling techniques, namely, Latent Dirichlet Allocation, Latent Semantic Analysis, Non-Negative Matrix Factorization, Principal Component Analysis, Random Project, K-Means, Top2Vec, BERT + K-Means, BERT, and Latent Dirichlet Allocation + BERT to determine the appropriate topic modeling approach. Topic diversity (indicating the model’s ability to capture differences between generated topics), coherence (the frequency of descriptive words of the topic within each cluster), and quality (topic diversity multiplied by topic coherence) were used as metrics to evaluate the effectiveness of isolating topics from the texts [41,42]. Finally, we used BERTopic to extract quality topics from the data based on the modelling performance. All texts were passed through the BERT model to create embeddings that converted text messages into numerical representations by using BERTopic. The dimensions of the embedding vectors were reduced due to the “Curse of Dimensionality” [43]. The bisecting K-means algorithm was used to group the reduced-dimension embedding vectors into clusters until optimal silhouette scores were achieved [44]. Various numbers of topics, ranging from 5 to 25, were tested to determine the optimal metrics.

Following the identification of clusters with the highest topic quality, the 20 most frequent words from each topic were extracted. BERTopic modeling was conducted using the Worcester Polytechnic Institute’s Turing cluster, which has high computing power for large volumes of data processing by using multiple physical computers simultaneously. The Turing cluster consists of a 4-node hyperconverged head that controls 79 compute nodes and is located at Worcester Polytechnic Institute. The total central processing unit/random access memory/graphics processing unit counts across all computer nodes were 5224, 49 TB, and 84, respectively [45].

### Sentiment and Thematic Analysis

Sentiment analysis was performed using 2 popular libraries, VADER (Valence Aware Dictionary and Sentiment Reasoner) and TextBlob [46,47]. Both libraries were used to enhance the accuracy of the results and minimize potential biases that may arise from relying on a single library. The sentiment scores obtained from each library were then averaged to derive a final sentiment score, which ranged from –1 (indicating a very negative sentiment) to 1 (indicating a very positive sentiment). Based on the topics generated through BERTopic modeling, thematic analysis was conducted by 2 investigators (XW and CSL) to identify themes associated with immunotherapy. The thematic analysis in this study involved the following steps: familiarization with the posts, inductive analysis, theme generation, and theme review among domain experts in the research team (including oncologists and oncology pharmacists) [48]. First, 2 investigators (XW and CSL) familiarized with the posts and encoded them independently. Then, the codes were compared, the same codes were merged, and any discrepancies were addressed through discussion with a third investigator (YTC). The themes were generated through the identification and refinement of the codes, which were grouped by perceived thematic similarities. The final themes were identified by achieving consensus among domain experts. The consensus process involved multiple rounds. The experts rated their level of agreement for each theme: themes with 75% agreement were retained, and themes without consensus were discussed, adjusted, and rated in the next round until 75% agreement was reached.

## Results

### Overview

A total of 4.9 million posts were gathered from online forums and social media platforms. After data cleaning, 3.6 million posts remained for further analysis (Figure 1).

### Number and Quality of the Topics

**Figure 2:**
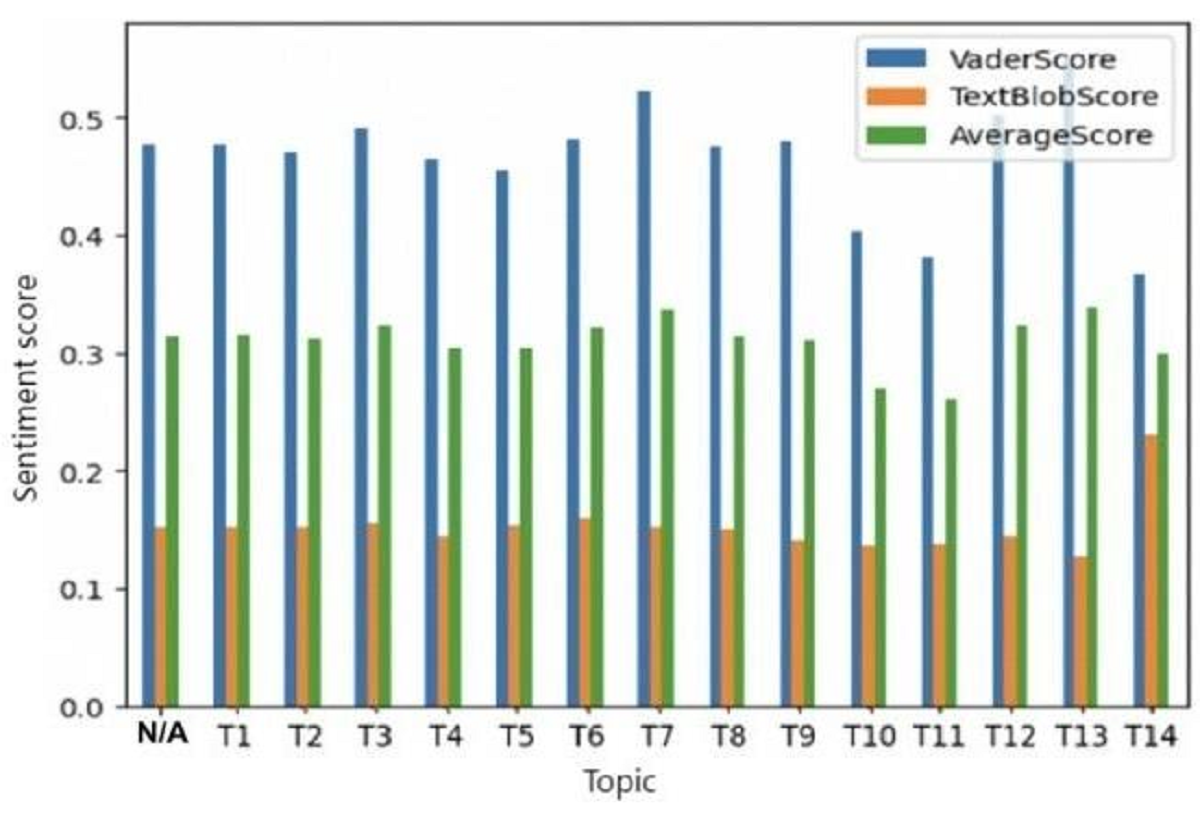
Sentiment analysis results. Sentiment score (a measure of how positive or negative the tone of the quotes is) was analyzed for each topic. The sentiment score ranged from –1 (indicating a very negative sentiment) to 1 (indicating a very positive sentiment). N/A: not applicable; T: topic.

In comparison with other topic modeling techniques, BERTopic demonstrated the most satisfactory performance, with a topic diversity score of 81.11%, a topic coherence score of 80.48%, and a topic quality score of 65.28% (Table S2 in Multimedia Appendix 1). Table S3 in Multimedia Appendix 1 presents BERTopic’s performance across different numbers of topics to determine the optimal value. Fifteen topics achieved the highest overall topic quality, scoring 70.47% (with the highest topic diversity at 87.86% and a high topic coherence of 80.21%). We also used these metrics to assess the quality of individual topics generated by the model, yielding topic quality scores ranging from 78% to 88% (Figure S1 in Multimedia Appendix 2). One topic (Topic 15) was excluded due to the mismatch between its extracted words and the topics related to immunotherapy. Based on the sentiment analysis results, the average sentiment score of around 0.3 across the 14 topics preliminarily suggested generally positive sentiments toward immunotherapy within the online community (Figure 2). A more comprehensive interpretation of the specific topics is presented in the subsequent sections.

### Themes and Topics

A total of 6 themes were identified (Table 2). The quotes in Table 2 have been modified to prevent any potential identification and direct linkage to the original posts. Theme 1 centered on the feelings of hope and positivity among patients treated with immunotherapy. This theme highlighted the belief that immunotherapy offers hope for patients with cancer to lead normal lives and provides a potential cure for cancer with minimal side effects. Theme 2 described immunotherapy as a more effective treatment option after other treatments have failed. Patients may have tried other cancer treatments such as chemotherapy and radiotherapy but only found successful results with immunotherapy. Besides, patients believe combining immunotherapy with other conventional therapies, particularly chemotherapy, can lead to higher efficacy in treating cancer than using a single treatment approach. Theme 3 suggested that patients receiving immunotherapy also explore diets or complementary medicines to manage their symptoms or side effects from treatment or the cancer itself. For example, patients may employ mind-body practices such as massage and meditation or use herbal medicines to alleviate certain physical and psychological side effects caused by immunotherapy. Probiotics were frequently mentioned by users as a potential way to boost the effectiveness of immunotherapy. In Theme 4, the potential ineffectiveness, worse outcomes, and high cost of treatment were frequently mentioned among patients, which acted as hindrances to the use of immunotherapy. Theme 5 suggested that immunotherapy can disrupt patients’ normal daily lives, particularly for those enrolled in immunotherapy trials. Additionally, the treatment may negatively impact their activities of daily living, such as taste changes, dietary restrictions, and time consumption. Theme 6 indicated that immunotherapy may cause physical and psychological symptoms and side effects such as rash, inflammation, and nephrotoxicity. These adverse effects may sometimes pose greater challenges compared to those arising from other treatments or the cancer itself. Some patients acknowledged that these symptoms may be unavoidable alongside the curative effects of immunotherapy.

**Table 2:** Topics and selected posts under each theme.

Theme, topics and related keywords		Samples of modified quotes^a^
**Theme 1: Hopeful prospects offered by immunotherapy**		
	Topic 1: good, time, hope, love, great, feel, work, lot, treatment, start	…help not to feel alone during treatment. …hope stop the growth of cancer quickly. …the new treatment offers new hope.
	Topic 3: fight, hope, positive, news, treatment, glad, beat, good, feel, life	…immunotherapy through clinical trials has shown positive results. …wish to continue with immunotherapy after completing treatment. …use the inert immune system to combat cancer cells. …reduced damage compared with chemotherapy or radiotherapy.
	Topic 5: hope, day, love, morning, huge, satisfy, glad, feel, good, love	…hope of one day curing all cancers without side effects. …develop immunotherapy tailored to the individual. …the day when immunotherapy will be available to all. …eradicate unique cancer cells
	Topic 6: saint, faith, family, love, god, strength, miracle, wonderful, hope, glad	…a miracle for patients to keep the cancer at bay. …a miracle to help someone with very advanced diseases.
**Theme 2: Perceived effectiveness of immunotherapy**		
	Topic 4: gallbladder, liver, bile, symptom, surgery, scan, remove, problem, ultrasound, test	…a tremendous response with significant improvements. …the tumors on my adrenal glands, lungs and liver have shrunk after immunotherapy. …tried immunotherapy as one last hail Mary pass and it seems to be working.
	Topic 13: risk, study, benefit, favorable, evidence, effect, patient, data, high, treat	…immunotherapy appears to be successful in that the CT scan shows the cancer has shrunk. …have a favorable response against the melanoma. …immunotherapy has given lives back.
	Topic 8: antioxidant, radiation, chemo, protect, treatment, dose, effect, radical, kill, interfere	…better results with less side effects. …combine chemo with immunotherapy gives the potential for long term remission and even a cure for advanced cancers. …very effective in treating liver tumors.
**Theme 3: Complementary therapies or self-treatments**		
	Topic 2: acupuncture, pain, feel, needle, massage, neuropathy, flash, hot, session, side	…encouraged to get massage and acupuncture. …had acupuncture to help with side effects of immunotherapy, such as abdominal pain, muscle pain, and severe neuropathy.
	Topic 10: keto, diet, food, organic, probiotic, supplement, eat, weight, sugar, body	…dependent on good gut bacteria. …anyone undergoing immunotherapy is recommended to take probiotics concurrently. …tried anything else, eg, diet, nutrition, supplements, etc.
**Theme 4: Financial and mental impact of immunotherapy**		
	Topic 7: diagnosis, feel, anxiety, traumatic, stress, cost, experience, time, depression, therapist	…expensive drugs with nasty side effects. …immunotherapy costs a lot that can’t afford it. …dealing with progression is profoundly stressful. …more depressed and weaker when waiting long for treatment.
**Theme 5: Impact on lifestyle and time schedules**		
	Topic 9: simulation, schedule, treatment, appointment, ill, week, start, time, plan, session	…involve an eight-week program after the previous treatments. …take a break and/or reduce dosages rather than quit altogether. …spent 5 weeks to decide on a new treatment plan. …don’t have too much flexibility.
**Theme 6: Side effects due to treatment**		
	Topic 11: ill, swell, weak, surgery, compression, feel, wear, inflame, tight, back	…surprised at how the side effects returned so quickly. …resulted in inflammation of lungs. …the treatments can result in worst side effects than the disease itself.
	Topic 12: immune, damage, skin, fever, burn, kill, system, therapy, tissue, body	…developed an acute kidney injury. …had a very hard time with auto immune reaction …had several other side effects, but not a rash.
	Topic 14: pain, symptom, hope, painful, colitis, arthritis, response, issue, experience, costochondritis	…back pain mentioned as a side effect. …have a rash on the back of neck and serious neuropathy. …found a cream that helps along with pain meds.

^a^The quotes have been modified or paraphrased to prevent any potential identification and direct linkage to the original posts.

## Discussion

### Principal Findings

We analyzed a large dataset comprising 3.6 million posts from 3 social media platforms and 16 online cancer forums, providing a comprehensive overview of perceptions regarding immunotherapy within English-speaking online cancer communities. A cutting-edge natural language processing technique, BERTopic, was used to generate interpretable topics, ensuring robust data analysis. The identification of different themes underscores the diverse attitudes toward immunotherapy across various aspects. The posts show the positive attitudes of the patients toward immunotherapy and its perceived effectiveness and benefits. However, there are also concerns about the side effects of immunotherapy and potential disruptions to patients’ lifestyle. These themes may offer valuable insights into the facilitators and barriers to the adoption of immunotherapy. In addition, the results showed that using machine learning techniques to analyze patient-generated online textual data can enhance our understanding of perspectives on newer cancer treatments, and such information may facilitate communication between patients and clinicians when discussing immunotherapy as a therapeutic option.

### Comparison to Current Evidence

The positive factors that encourage the utilization of immunotherapy included its comparatively mild side effect profile and the potential to instill hope in patients by offering a viable treatment option when other methods have failed. Our findings also indicated that online cancer communities seem to be well-informed, with some patients well-versed in the benefits of immunotherapy. Laypeople now access medical information on the internet independently and acquire firsthand information about advances in cancer treatment. Access to online health information assists patients in making health-related decisions, leading to more professional consultation, improved treatment compliance, and better self-care [49]. However, the internet should not be viewed as a replacement for professional health information sources [50]. The guidance and advice of health care professionals remain indispensable in helping patients make complex medical decisions [51]. Online health information cannot provide detailed diagnoses or personalized treatment plans for patients; moreover, the presence of misinformation on websites might mislead patients about treatment [52]. Taken together, our results highlight the rising health literacy of online cancer communities, especially those involving younger generations. Future efforts should focus on enhancing the accuracy of online health information by using reliable rating tools, effective search engine ranking, and progress in crowdsourcing websites.

Although immunotherapy has shown clinical benefits in several trials, there are still potential barriers to its utilization. The high cost associated with immunotherapy emerges as a frequently discussed concern among patients with cancer, emphasizing the need for revising drug valuations and reimbursement models [53]. Many quotes were related to patients’ descriptions of their physical symptoms induced by immunotherapy and how it has impeded their activities of daily living and disrupted their normal lives. In addition to physical irAEs, the long-term use of immunotherapy has also been associated with psychiatric side effects such as fatigue, insomnia, anxiety, and depression [54,55]. Hence, it is important for oncology practitioners to manage patients’ expectations and communicate with patients regarding the potential side effects of immunotherapy before initiating treatment. This proactive approach allows patients to better understand the benefits and risks of the treatment, which may prevent them from discontinuing treatment because of known adverse reactions.

### Relevance to Clinical Practice

Several clinical practice guidelines have been proposed to address the evaluation and management of immunotherapy-induced toxicity [56] and the diagnosis and treatment of toxicity associated with immune checkpoint inhibitor therapy in specific organ systems [57]. The provision of high-quality supportive care from a multidisciplinary team of health care professionals can help identify and manage irAEs [58]. In practice, patients may turn to self-management strategies to cope with these symptoms. We found that some patients mentioned in online posts that they used traditional, complementary, and integrative medicine modalities such as probiotics, herbal remedies, and meditation to self-manage the toxicity induced by immunotherapy. Recently, some studies have evaluated how complementary medicines may enhance the efficacy of immunotherapy and alleviate immune toxicity [59,60]. For example, the treatment of irAEs often necessitates the long-term use of high-dose corticosteroids [61]. Integrating complementary modalities into the management of irAEs may reduce corticosteroid use. Our group has previously reported that patients with cancer frequently seek advice on the use of complementary medicines on social media platforms [20]. At this juncture, clinicians should focus on initiating effective communication regarding the use of complementary medicines and help patients establish realistic expectations while being well-informed about the limited evidence supporting these approaches.

### Limitations

Our study has several limitations. First, the data used in this research were collected from English-language online cancer communities, which may limit the generalizability of the findings to data posted in other languages. Social media and cancer forums may be used distinctly in different countries, and the types of immunotherapeutic approaches may also vary among regions. Future studies could conduct a thorough investigation into whether distinct concerns regarding immunotherapy exist among patients from different countries and regions of practice. Second, perceptions regarding immunotherapy on social media and cancer forums are dynamic and may be influenced by current news or events. Therefore, future research can consider exploring the fluctuations in posts on these platforms over varying time periods. Third, although the performance metrics (topic diversity and topic coherence scores) quantified from our models suggest that BERTopic is effective at unveiling latent patterns within large datasets, this approach requires validation. Nevertheless, our study demonstrates that applying artificial intelligence to analyze social media data can facilitate our understanding of patients’ perspectives as well as the effectiveness of machine-learning techniques in processing substantial volumes of data retrieved from social media platforms and online forums.

### Conclusions

This study captures comprehensive insights and perspectives of online communities regarding immunotherapy through BERTopic modeling. The identified themes and topics may facilitate the understanding of immunotherapy utilization and serve as valuable supporting information when making treatment decisions. The posts have shown the positive attitudes of the patients and their perceived effectiveness and benefits of immunotherapy. Although immunotherapy presents a beacon of hope and a viable treatment option, its side effects and the accompanying potential lifestyle disruptions cannot be ignored and are major concerns perceived by the online communities. It is essential for clinicians to inform patients of the undesirable effects of immunotherapy and establish realistic expectations, thereby enabling patients to make an informed decision considering the benefits and risks associated with the treatment, which may ultimately lead to more optimal treatment outcomes with immunotherapy.

## Appendix

Multimedia Appendix 1 Additional data.

Multimedia Appendix 2 The distribution of topic quality scores.

## Abbreviations

BERT: Bidirectional Encoder Representation from Transformers
BERTopic: a topic modeling technique that is an extension of the Bidirectional Encoder Representation from Transformers
irAE: immunotherapy-related adverse event
VADER: Valence Aware Dictionary and Sentiment Reasoner

## Data Availability

The dataset used in this study is accessible via the following link [62].

## References

[1] (2019). Immunotherapy to treat cancer. National Cancer Institute.

[2] Miller KD, Nogueira L, Devasia T, Mariotto AB, Yabroff KR, Jemal A, Kramer J, Siegel RL (2022). Cancer treatment and survivorship statistics, 2022. CA Cancer J Clin.

[3] Tan S, Li D, Zhu X (2020). Cancer immunotherapy: Pros, cons and beyond. Biomed Pharmacother.

[4] Chen DS, Mellman I (2017). Elements of cancer immunity and the cancer-immune set point. Nature.

[5] Bien DR, Danner M, Vennedey V, Civello D, Evers SM, Hiligsmann M (2017). Patients' preferences for outcome, process and cost attributes in cancer treatment: a systematic review of discrete choice experiments. Patient.

[6] Ihrig A, Richter J, Bugaj TJ, Friederich H, Maatouk I (2023). Between hope and reality: How oncology physicians and information providers of a cancer information service manage patients' expectations for and experiences with immunotherapies. Patient Educ Couns.

[7] Hesse BW, Greenberg AJ, Rutten LJF (2016). The role of internet resources in clinical oncology: promises and challenges. Nat Rev Clin Oncol.

[8] Iftikhar R, Abaalkhail B (2017). Health-seeking influence reflected by online health-related messages received on social media: cross-sectional survey. J Med Internet Res.

[9] Jenei K, Burgess M, Peacock S, Raymakers AJN (2021). Experiences and perspectives of individuals accessing CAR-T cell therapy: A qualitative analysis of online Reddit discussions. J Cancer Policy.

[10] Booth A, Manson S, Halhol S, Merinopoulou E, Raluy-Callado M, Hareendran A, Knoll S (2023). Using health-related social media to understand the experiences of adults with lung cancer in the era of immuno-oncology and targeted therapies: observational study. JMIR Cancer.

[11] Renner S, Loussikian P, Foulquié P, Marrel A, Barbier V, Mebarki A, Schück S, Bharmal M (2023). Patient and caregiver perceptions of advanced bladder cancer systemic treatments: infodemiology study based on social media data. JMIR Cancer.

[12] Rodrigues A, Chauhan J, Sagkriotis A, Aasaithambi S, Montrone M (2022). Understanding the lived experience of lung cancer: a European social media listening study. BMC Cancer.

[13] Chang A, Xian X, Liu MT, Zhao X (2022). Health communication through positive and solidarity messages amid the COVID-19 pandemic: automated content analysis of Facebook uses. Int J Environ Res Public Health.

[14] Grootendorst M (2022). BERTopic: Neural topic modeling with a class-based TF-IDF procedure. ArXiv.

[15] Ng QX, Lee DYX, Yau CE, Lim YL, Liew TM (2023). Public perception on 'healthy ageing' in the past decade: An unsupervised machine learning of 63,809 Twitter posts. Heliyon.

[16] Ng QX, Yau CE, Lim YL, Wong LKT, Liew TM (2022). Public sentiment on the global outbreak of monkeypox: an unsupervised machine learning analysis of 352,182 twitter posts. Public Health.

[17] Devlin J, Chang M, Lee K, Toutanova K (2019). Bert: Pre-training of deep bidirectional transformers for language understanding. https://aclanthology.org/N19-1423/.

[18] Nayak P Understanding searches better than ever before. Google.

[19] Wagner J The most popular social networks of 2021. Ignite Social Media.

[20] Lam CS, Zhou K, Loong HH, Chung VC, Ngan C, Cheung YT (2023). The use of traditional, complementary, and integrative medicine in cancer: data-mining study of 1 million web-based posts from health forums and social media platforms. J Med Internet Res.

[21] Search engine market share worldwide. Statcounter GlobalStats.

[22] Cancer chat. Cancer Research UK.

[23] Jo's Cervical Cancer Trust Forum.

[24] Prostate Cancer UK.

[25] Bowel Cancer UK.

[26] Cancer Survivors Network.

[27] The Breastcancer.org Community Forum.

[28] Pancreatic Cancer UK.

[29] Breast Cancer Now Forum.

[30] Cancer Council Online Community.

[31] Irish Cancer Society Community.

[32] Navigating Care.

[33] Testicular Cancer Society.

[34] HealthBoards (cancer).

[35] Melanoma Patient Forum.

[36] Macmillan Cancer Support.

[37] HealthUnlocked.

[38] Kocaman V, Talby D (2021). Spark NLP: Natural language understanding at scale. Software Impacts.

[39] Health care NLP state of the art medical language models. John Snow Labs.

[40] Saracco BH (2020). Data science and predictive analytics: biomedical and health applications using R. Journal of the Medical Library Association.

[41] Dieng AB, Ruiz FJR, Blei DM (2020). Topic modeling in embedding spaces. Transactions of the Association for Computational Linguistics.

[42] Mimno D, Wallach H, Talley E, Leenders M, McCallum A (2011). Optimizing semantic coherence in topic models. https://aclanthology.org/D11-1024.

[43] Grootendorst M (2022). BERTopic.

[44] Krishna BSV, Satheesh P, SuneelKumar R (2012). Comparative study of k-means and bisecting k-means techniques in wordnet based document clustering. Semantic Scholar.

[45] High performance computing. Worcester Polytechnic Institute.

[46] Bonta V, Kumaresh N, Janardhan N (2019). A comprehensive study on lexicon based approaches for sentiment analysis. AJCST.

[47] Aljedaani W, Rustam F, Mkaouer MW, Ghallab A, Rupapara V, Washington PB, Lee E, Ashraf I (2022). Sentiment analysis on Twitter data integrating TextBlob and deep learning models: The case of US airline industry. Knowledge-Based Systems.

[48] Gonzalez G, Vaculik K, Khalil C, Zektser Y, Arnold C, Almario CV, Spiegel B, Anger J (2022). Using large-scale social media analytics to understand patient perspectives about urinary tract infections: thematic analysis. J Med Internet Res.

[49] Thapa DK, Visentin DC, Kornhaber R, West S, Cleary M (2021). The influence of online health information on health decisions: A systematic review. Patient Educ Couns.

[50] Jacobs W, Amuta AO, Jeon KC (2017). Health information seeking in the digital age: An analysis of health information seeking behavior among US adults. Cogent Social Sciences.

[51] Palanica A, Flaschner P, Thommandram A, Li M, Fossat Y (2019). Physicians' perceptions of chatbots in health care: cross-sectional web-based survey. J Med Internet Res.

[52] Suarez-Lledo V, Alvarez-Galvez J (2021). Prevalence of health misinformation on social media: systematic review. J Med Internet Res.

[53] Tran G, Zafar SY (2018). Financial toxicity and implications for cancer care in the era of molecular and immune therapies. Ann Transl Med.

[54] Kovacs D, Kovacs P, Eszlari N, Gonda X, Juhasz G (2016). Psychological side effects of immune therapies: symptoms and pathomechanism. Curr Opin Pharmacol.

[55] Lacouture M, Sibaud V (2018). Toxic side effects of targeted therapies and immunotherapies affecting the skin, oral mucosa, hair, and nails. Am J Clin Dermatol.

[56] Haanen J, Obeid M, Spain L, Carbonnel F, Wang Y, Robert C, Lyon AR, Wick W, Kostine M, Peters S, Jordan K, Larkin J, ESMO Guidelines Committee. Electronic address: clinicalguidelines@esmo.org (2022). Management of toxicities from immunotherapy: ESMO Clinical Practice Guideline for diagnosis, treatment and follow-up. Ann Oncol.

[57] Brahmer JR, Lacchetti C, Schneider BJ, Atkins MB, Brassil KJ, Caterino JM, Chau I, Ernstoff MS, Gardner JM, Ginex P, Hallmeyer S, Holter Chakrabarty J, Leighl NB, Mammen JS, McDermott DF, Naing A, Nastoupil LJ, Phillips T, Porter LD, Puzanov I, Reichner CA, Santomasso BD, Seigel C, Spira A, Suarez-Almazor ME, Wang Y, Weber JS, Wolchok JD, Thompson JA, National Comprehensive Cancer Network (2018). Management of immune-related adverse events in patients treated with immune checkpoint inhibitor therapy: American Society of Clinical Oncology Clinical Practice Guideline. J Clin Oncol.

[58] Rahman MM, Behl T, Islam MR, Alam MN, Islam MM, Albarrati A, Albratty M, Meraya AM, Bungau SG (2022). Emerging management approach for the adverse events of immunotherapy of cancer. Molecules.

[59] Zhang N, Xiao X (2021). Integrative medicine in the era of cancer immunotherapy: Challenges and opportunities. J Integr Med.

[60] Jia W, Wang L (2020). Using Traditional Chinese Medicine to treat hepatocellular carcinoma by targeting tumor immunity. Evid Based Complement Alternat Med.

[61] Schneider BJ, Naidoo J, Santomasso BD, Lacchetti C, Adkins S, Anadkat M, Atkins MB, Brassil KJ, Caterino JM, Chau I, Davies MJ, Ernstoff MS, Fecher L, Ghosh M, Jaiyesimi I, Mammen JS, Naing A, Nastoupil LJ, Phillips T, Porter LD, Reichner CA, Seigel C, Song J, Spira A, Suarez-Almazor M, Swami U, Thompson JA, Vikas P, Wang Y, Weber JS, Funchain P, Bollin K (2021). Management of immune-related adverse events in patients treated with immune checkpoint inhibitor therapy: ASCO guideline update. JCO.

[62] Cheung YT Perception of online communities towards the use of cancer immunotherapy: a data mining study of 3.6 million web-based posts from social media platforms using BERTopic. The Chinese University of Hong Kong.

