# Supplementary material for: Perceptions in 3.6 Million Web-Based Posts of Online Communities on the Use of Cancer Immunotherapy: Data Mining Using BERTopic
Source: J Med Internet Res. 2025 Feb 10;27:e60948. doi: 10.2196/60948 (PMC11851037; doi:10.2196/60948)
Supplement: Multimedia Appendix 1 [file jmir_v27i1e60948_app1.docx]

Table S1. Example of keywords related to immunotherapy for post/thread search and the data sources.

| Keywords |
| --- |
| Immune checkpoint inhibitor and monoclonal antibodies  Immune checkpoint inhibitor, Immune checkpoint modulator, Atezolizumab, Avelumab, Cemiplimab, Checkpoint inhibitor, Checkpoint modulator, Dostarlimab, Durvalumab, Ipilimumab, Nivolumab, Pembrolizumab, Bavencio, Libtayo, Jemperli, Imfinzi, Keytruda, Opdivo, Tecentriq, Yervoy, PD-1, PD-L1, CTLA4, CTLA-4, Cytotoxic T-lymphocyte-associated protein 4, Programmed cell death protein 1, Programmed death-ligand 1, Relatlimab, Retifanlimab, Zynyz, Tremelimumab, Imjudo |
| Immune system modulators  Interferon, IFN, Pegasys, PegIFN, Peginterferon, Pegylated interferon, Proleukin, Roferon, Intron, Avonex, Betaferon, Aldesleukin, Immune modulator, Lenalidomide, Pomalidomide, Thalidomide, Rebif, CSF, GM-CSF, Colony Stimulating Factor, Imnovid, Pomalyst, Revlimid |
| Monoclonal antibodies  Alemtuzumab, Bevacizumab, Brentuximab, Burosumab, Cetuximab, Daratumumab, Elotuzumab, Gemtuzumab, Ibritumomab, Inotuzumab, Obinutuzumab, Panitumumab, Pertuzumab, Polatuzumab, Ramucirumab, Rituximab, Siltuximab, Trastuzumab, Campath, Avastin, Adcetris, Crysvita, Cyranza, Darzalex, Empliciti, Erbitux, Herceptin, Gazyvaro, Besponsa, Mabthera, Mylotarg, Perjeta, Polivy, Sylvant, Vectibix, Zevalin, Blinatumomab, Blincyto, Lemtrada, Isatuximab, Sarclisa, Margetuximab, Margenza, Mogamulizumab, Poteligeo, Naxitamab, Danyelza, Necitumumab, Portrazza, Ofatumumab, Arzerra, Olaratumumab, Latruvo, Tafasitamab, Monjuvi, Enfortumab, Padcev, Loncastuximab, Zynlonta, Mirvetuximab, Elahere, Moxetumomab, Lumoxiti, Sacituzumab, Trodelvy, Tisotumab, Tivdak, Amivantamab, Rybrevant, Epcoritamab, Epkinly, Glofitamab, Columvi, Mosunetuzumab, Lunsumio, Tebentafusp, Kimmtrak, Tecvayli, Teclistimab, Monoclonal |
| Adoptive cell therapies  CAR T cell, Chimeric Antigen Receptor T cells, Tisagenlecleucel, Axicabtagene, Brexucabtagene, Yescarta, Tecartus, Kymriah, Adoptive cell therapy, Adoptive T Cell Transfer, Tumor-infiltrating lymphocyte, Ciltacabtagene, Carvykti, Idecabtagene, Abecma, Isocabtagene, Breyanzi |
| Others  Oncolytic virus, lmlygic, T-vec, Talimogene, Cancer vaccine, BCG, Bacillus Calmette-Guerin, HPV, Papillomavirus, Provenge, Spuleucel, Cervarix, Gardasil, Immunotherapy, Immuno-oncology |
| Data sources |
| Cancer Research UK  <https://www.cancerresearchuk.org/about-cancer/treatment/immunotherapy/what-is-immunotherapy>  American Cancer Society  <https://www.cancer.org/cancer/managing-cancer/treatment-types/immunotherapy.html>  Memorial Sloan Kettering Cancer Centre  <https://www.mskcc.org/cancer-care/diagnosis-treatment/cancer-treatments/immunotherapy>  Cancer.Net® (American Society of Clinical Oncology)  <https://www.cancer.net/navigating-cancer-care/how-cancer-treated/immunotherapy-and-vaccines/what-immunotherapy>  National Cancer Institute  <https://www.cancer.gov/about-cancer/treatment/types/immunotherapy> |

Table S2. The performances of ten topic modeling methods.

| Methods | Time to Fit | Topic Diversity | Topic Coherence | Topic Quality |
| --- | --- | --- | --- | --- |
| LDA | 15m | 25.00% | 81.61% | 20.40% |
| LSA | 1h 23m | 35.00% | 83.37% | 29.18% |
| NMF | 25m | 40.00% | 80.86% | 32.34% |
| PCA | 3m | 60.00% | 81.57% | 48.94% |
| Random Project | 2m | 17.00% | 80.66% | 13.71% |
| K-Means | 36m | 37.00% | 80.36% | 29.73% |
| Top2Vec | 2h 9m | 76.00% | 73.15% | 55.59% |
| BERT + K-Means | 7h 29m | 33.00% | 80.71% | 26.63% |
| LDA + BERT | 22h 1m | 36.00% | 80.51% | 28.98% |
| BERTopic | 7h 43m | 81.11% | 80.48% | 65.28% |

Notes: LDA, Latent Dirichlet Allocation; LSA, Latent Semantic Analysis; NMF, Non-negative Matrix Factorization; PCA, Principal Component Analysis; BERT, Bidirectional Encoder Representation from Transformers.

Table S3. The performances of BERTopic on different numbers of topics.

| Topic Count | Time to Fit | Topic Diversity | Topic Coherence | Topic Quality |
| --- | --- | --- | --- | --- |
| 5 | 14h 42m | 87.50% | 76.74% | 67.15% |
| 10 | 7h 43m | 81.11% | 80.48% | 65.28% |
| 12 | 6h 10m | 84.55% | 80.19% | 67.80% |
| 13 | 6h 20m | 82.50% | 80.20% | 66.17% |
| 14 | 9h 54m | 82.31% | 79.57% | 65.49% |
| 15 | 9h 27m | 87.86% | 80.21% | 70.47% |
| 16 | 8h 53m | 70.00% | 82.84% | 57.99% |
| 17 | 10h 12m | 77.50% | 81.76% | 63.36% |
| 18 | 13h 43m | 78.24% | 80.61% | 63.07% |
| 20 | 7h 54m | 75.26% | 79.09% | 59.52% |
| 25 | 9h 49m | 76.67% | 78.34% | 60.06% |
