# Supplementary figures and images for: Perceptions in 3.6 Million Web-Based Posts of Online Communities on the Use of Cancer Immunotherapy: Data Mining Using BERTopic
Source: J Med Internet Res. 2025 Feb 10;27:e60948. doi: 10.2196/60948 (PMC11851037; doi:10.2196/60948)

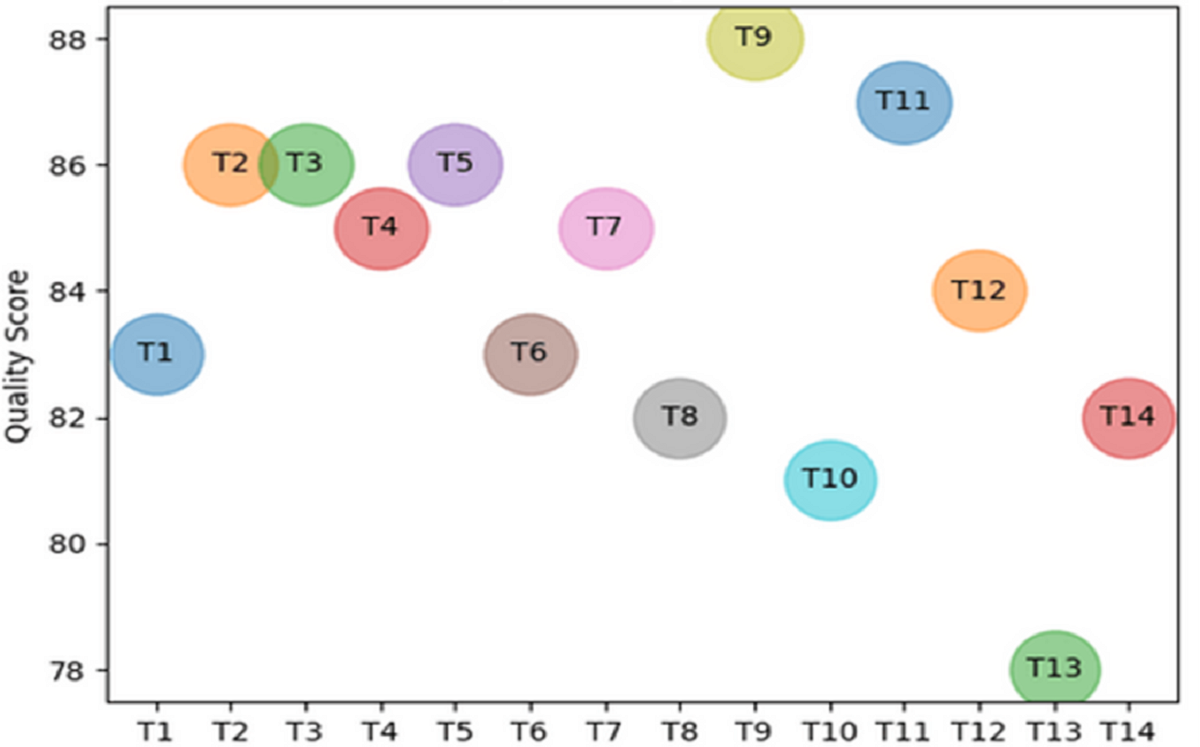

Supplement: Multimedia Appendix 2 [file jmir_v27i1e60948_app2.png]
